# A CRISPR Interference Platform for Efficient Genetic Repression in *Candida albicans*

**DOI:** 10.1128/mSphere.00002-19

**Published:** 2019-02-13

**Authors:** Lauren Wensing, Jehoshua Sharma, Deeva Uthayakumar, Yannic Proteau, Alejandro Chavez, Rebecca S. Shapiro

**Affiliations:** a Department of Molecular and Cellular Biology, University of Guelph, Guelph, Ontario, Canada; b Department of Pathology and Cell Biology, Columbia University College of Physicians and Surgeons, New York, New York, USA; Carnegie Mellon University

**Keywords:** CRISPR, CRISPRi, *Candida*, *Candida albicans*, fungal genetics, genetic regulation, genetic technology

## Abstract

Fungal pathogens are emerging as an important cause of human disease, and Candida albicans is among the most common causative agents of fungal infections. Studying this fungal pathogen is of the utmost importance and necessitates the development of molecular technologies to perform comprehensive genetic and functional genomic analysis. Here, we designed and developed a novel clustered regularly interspaced short palindromic repeat interference (CRISPRi) system for targeted genetic repression in C. albicans. We engineered a nuclease-dead Cas9 (dCas9) construct that, paired with a guide RNA targeted to the promoter of an endogenous gene, is capable of targeting that gene for transcriptional repression. We further optimized a favorable promoter locus to achieve repression and demonstrated that fusion of dCas9 to an Mxi1 repressor domain was able to further enhance transcriptional repression. Finally, we demonstrated the application of this CRISPRi system through genetic repression of the essential molecular chaperone *HSP90*. This is the first demonstration of a functional CRISPRi repression system in C. albicans, and this valuable technology will enable many future applications in this critical fungal pathogen.

**IMPORTANCE** Fungal pathogens are an increasingly important cause of human disease and mortality, and Candida albicans is among the most common causes of fungal disease. Studying this important fungal pathogen requires a comprehensive genetic toolkit to establish how different genetic factors play roles in the biology and virulence of this pathogen. Here, we developed a CRISPR-based genetic regulation platform to achieve targeted repression of C. albicans genes. This CRISPR interference (CRISPRi) technology exploits a nuclease-dead Cas9 protein (dCas9) fused to transcriptional repressors. The dCas9 fusion proteins pair with a guide RNA to target genetic promoter regions and to repress expression from these genes. We demonstrated the functionality of this system for repression in C. albicans and show that we can apply this technology to repress essential genes. Taking the results together, this work presents a new technology for efficient genetic repression in C. albicans, with important applications for genetic analysis in this fungal pathogen.

## Abstract (precis)

Fungal pathogens are an increasingly important cause of human disease and mortality, and Candida albicans is among the most common causes of fungal disease. Studying this important fungal pathogen requires a comprehensive genetic toolkit to establish how different genetic factors play roles in the biology and virulence of this pathogen. Here, we developed a CRISPR-based genetic regulation platform to achieve targeted repression of C. albicans genes. This CRISPR interference (CRISPRi) technology exploits a nuclease-dead Cas9 protein (dCas9) fused to transcriptional repressors. The dCas9 fusion proteins pair with a guide RNA to target genetic promoter regions and to repress expression from these genes. We demonstrated the functionality of this system for repression in C. albicans and show that we can apply this technology to repress essential genes. Taking the results together, this work presents a new technology for efficient genetic repression in C. albicans, with important applications for genetic analysis in this fungal pathogen.

## INTRODUCTION

Invasive fungal infections have emerged as an important cause of human mortality, particularly for an ever-increasing population of immunocompromised individuals (1–3). The rise in the incidence of these opportunistic invasive infections is associated with many factors, including the HIV/AIDS epidemic and the growing number of patients receiving immunosuppressive therapeutics for bone marrow and organ transplantations or for the treatment of autoimmune disorders (4). Invasive fungal infections are associated with disproportionately high rates of patient mortality (∼30% to 90% mortality, depending on the pathogen and patient group [5, 6]), and with a massive economic burden (∼$7.2 billion in 2017) (4, 7, 8). Among these fungal pathogens, *Candida* species are among those representing the most common causes of infections, accounting for ∼55% of invasive fungal infections in North America (4). Candida albicans is the leading cause of invasive candidiasis and a leading cause of nosocomial bloodstream infection (4). C. albicans is a polymorphic yeast species which exists as a commensal member of the human microbiota and as an opportunistic pathogen, able to cause disease ranging from relatively benign superficial infections to life-threatening invasive infections.

As a critically important human fungal pathogen, C. albicans has been subjected to in-depth molecular genetic analysis to uncover factors involved in its virulence, interactions with the host, resistance to antifungal agents, and other important biological processes. Previously, C. albicans was considered to be a highly intractable microbial organism, due to limitations associated with genetic manipulation, including an inability to stably maintain plasmids, an unusual form of codon usage (the CUG codon is translated as serine instead of leucine [9]), inefficient homologous recombination, and its diploid nature. However, in the last ∼10 years, new advances in functional genomic technologies, as well as the discovery of mating-competent C. albicans haploid strains (10), have enabled a growing number of large-scale functional genomic studies in this clinically relevant pathogen. This important research has included the development of new technologies for genetic manipulation in C. albicans, including genetic deletion systems (11, 12), conditional expression systems (13), double-selection-based deletion systems (14), and transposon mutagenesis platforms (15–17). These technologies have been applied in a variety of innovative ways to identify genetic factors underpinning C. albicans morphogenesis and biofilm formation (12, 18–21), fungus-host interactions (12, 22), and mechanisms of antifungal drug resistance (16, 23–25) and for the identification of essential genes (13, 15, 26).

Despite this existing research repertoire of functional genomic studies in C. albicans, new genetic tools continue to improve and refine our ability for targeted genetic analysis. One example of a genetic tool that has revolutionized targeted genetic manipulation in a diversity of fungal and other microbial species is clustered regularly interspaced short palindromic repeat (CRISPR)-based technology (27). Recently, CRISPR-based technologies have been applied for targeted genetic mutations and deletions in C. albicans (28–33), as well as in other closely related *Candida* species (34–36). Each of these systems relies on the foundational CRISPR editing system, whereby a Cas9 endonuclease pairs with a single guide RNA (sgRNA), comprising a Cas9-binding region (the conserved sgRNA “tail”) and a unique 20 nucleotide “N20” region complementary to the targeted genomic locus. This sgRNA-Cas9 complex interacts with a locus based on complementary binding of the sgRNA N20 to the target region, provided that a necessary protospacer adjacent motif (PAM) is also present within the target locus. After binding, the Cas9 endonuclease undergoes a conformational change, generating a double-stranded break (DSB) within the DNA region (37). This DSB can then be repaired via nonhomologous end joining (NHEJ) or via homology-directed repair when repair donor DNA with homology to the region surrounding the DSB is provided. The latter mechanism is what has been most commonly exploited for CRISPR-based genetic manipulation in *Candida* and other yeast species (28–36, 38).

Since their development as genetic editing technologies (39), CRISPR systems have been further modified to achieve alternative outcomes, such as base-editing (40–42), RNA editing (43), epigenetic modifications (44, 45), and transcriptional regulation (46). CRISPR transcriptional repression relies on a precisely mutated, nuclease-dead version of the Cas9 endonuclease (dCas9), which is targeted to specific genomic promoter regions by sgRNAs to achieve steric hindrance of RNA polymerase (Pol), thus blocking transcription initiation or elongation (46–49). CRISPR interference (CRISPRi)-based genetic repression was first demonstrated in mammalian cells and Escherichia coli (47) and has since been applied in a diversity of other microbial species (50–53). Fusing repressor domains to dCas9 can further enable transcriptional repression. For instance, the Krüppel associated box (KRAB) and MeCP2 transcriptional repression domain can be fused to dCas9 to significantly enhance target gene repression in human cells (52), and dCas9-Mxi1 fusions similarly enhance repression in Saccharomyces cerevisiae (52, 54). CRISPRi presents certain advantages in comparison to traditional CRISPR editing systems: it facilitates the study of essential genes, enables a titratable system to regulate the level of gene expression, is reversible, and is generally significantly easier to engineer, as it does not rely on homology-directed repair or on the presence of repair donor DNA templates. The CRISPRi framework can also be exploited for CRISPR activation (CRISPRa) by fusing dCas9 to activator domains, such as VP64, to drive transcriptional activation from a desired locus (55–57).

While CRISPR-based editing has been used to efficiently generate genetic mutations and deletions in *Candida* species, the functionality of other CRISPR technologies, such as CRISPRi and CRISPRa, has yet to be explored in these fungal pathogens. Here, we present the first report detailing the design and execution of a CRISPRi platform for genetic repression in C. albicans. Using C. albicans-optimized dCas9, we demonstrated that CRISPRi can be used to repress gene expression in C. albicans and further demonstrated that effector fusion constructs such as dCas9-Mxi1 can be used to achieve high levels of transcriptional repression (∼20-fold repression) for a target locus. Finally, we use this optimized CRISPRi dCas9-Mxi1 system to demonstrate the ability to repress expression of *HSP90*, the essential C. albicans molecular chaperone, and to recapitulate phenotypes associated with its genetic depletion. Taken together, the results reveal a novel genetic technology for efficient genetic repression in C. albicans, with important applications for functional genomic analysis in this critical fungal pathogen.

## RESULTS

### Design and preliminary validation of a CRISPRi system for C. albicans.

**FIG 1:**
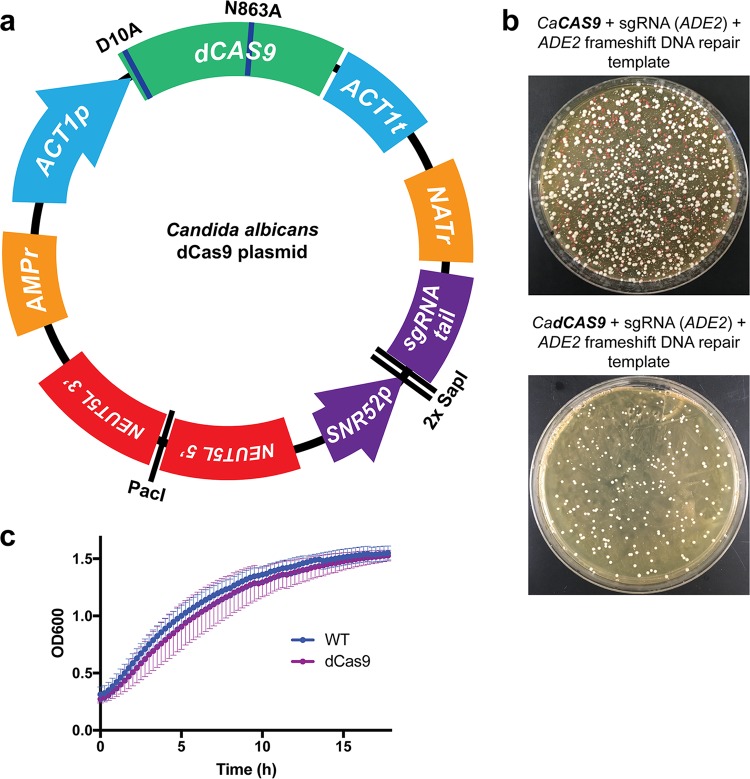
Design and validation of a CRISPRi system for C. albicans. (a) dCas9 plasmid engineered for CRISPRi repression. This dCas9-based plasmid represents an all-in-one system for CRISPRi repression in C. albicans. All components have been codon optimized for C. albicans, and two nuclease mutations (D10A and N863A) have been introduced into Cas9 to render it nuclease-dead (dCas9). *NEUT5L* homology is present for integration into the C. albicans genome upon plasmid linearization with PacI. The two SapI cloning sites allow simple sgRNA N20 cloning to generate unique sgRNAs. (b) dCas9 is deficient with respect to its nuclease function. Side-by-side comparisons of C. albicans transformation plates were performed using a Cas9 and dCas9 plasmid with sgRNAs targeting the *ADE2* ORF for Cas9-mediated DSB. The two strains were cotransformed with a repair donor DNA template harboring a frameshift mutation to generate a premature stop codon in the *ADE2* gene, leading to loss of function, and a red phenotype. Absence of observed red colonies upon transformation of the dCas9 construct suggests that it was deficient with respect to its nuclease activity. CaCAS9, C. albicans Cas9. (c) The dCas9 plasmid integrated in the C. albicans genome does not affect growth. Growth curves were performed using a wild-type C. albicans strain and one with the dCas9 plasmid integrated in its genome at the *NEUT5L* locus. The dCas9-containing strain did not show a defect in growth compared to the wild-type (WT) strain.

Initially, to develop a CRISPRi system for genetic repression in C. albicans, we generated a nuclease-dead version of Cas9 (dCas9), optimized for use in C. albicans. We exploited a plasmid backbone that we had previously used for successful CRISPR-based genetic deletions in C. albicans using Cas9 (33), modifying the C. albicans codon-optimized *CAS9* genes to contain two single nucleotide mutations (RuvC nuclease domain mutation D10A and HNH nuclease domain mutation N863A), previously associated with impairment of nuclease function in Cas9 (47, 58). We incorporated this dCas9 into a plasmid backbone to generate a single, “all-in-one” plasmid to facilitate CRISPRi regulation in C. albicans (Fig. 1a). This plasmid contains all the required components to achieve CRISPRi regulation in C. albicans and is readily modified to target any gene of interest (Fig. 1a). The critical elements of this plasmid include the following: (i) C. albicans-optimized dCas9; (ii) selection markers for bacteria (ampicillin resistance [AMPr]) and C. albicans (nourseothricin resistance [NATr]); (iii) regions of homology to the C. albicans *NEUT5L* locus to enable stable integration of the plasmid at this neutral locus (59) upon plasmid linearization with restriction enzyme PacI; and (iv) a sgRNA cloning locus, which contains two SapI restriction enzyme sites for efficient Golden Gate cloning (60) of unique N20 sgRNA sequences, between the SNR52 RNA polymerase III (Pol III) promoter used to drive sgRNA expression and the conserved sgRNA tail (Fig. 1a). This permits simple, Golden Gate cloning of unique N20 sequences into the dCas9 plasmid to target sgRNA-dCas9 to the promoter region of any gene of interest.

In order to assess whether the dCas9 construct was deficient in nuclease activity, we compared it directly to an equivalent plasmid harboring the nonmutated C. albicans-optimized *CAS9* gene. We designed a sgRNA N20 targeting within the C. albicans *ADE2* open reading frame (ORF) for Cas9-mediated cleavage, as well as a repair DNA template introducing a frameshift mutation into the *ADE2* ORF, thereby introducing a premature stop codon, and a loss-of-function allele of *ADE2*. The *ADE2*-targeting N20 sequence was ligated into both the Cas9 and dCas9 plasmids at the SapI sites, and the plasmids were transformed into C. albicans strains along with the repair DNA. As expected, we found that the Cas9 construct was able to mutate *ADE2*, based on the presence of red colonies on the transformation plate (Fig. 1b). However, the dCas9 construct was unable to cause double-strand breaks and thus was unable mutate *ADE2*, and produced no red colonies (Fig. 1b), indicating that dCas9 has in fact lost its nuclease activity.

Next, we assessed whether the dCas9 construct imparted any significant fitness defect to the C. albicans strains. We monitored growth of a wild-type C. albicans strain, compared to one harboring the dCas9 plasmid integrated at the *NEUT5L* locus. This dCas9 strain contains an irrelevant, nontargeting sgRNA (including the *SNR52* promoter and complete sgRNA with an sgRNA tail and an N20 that does not target the C. albicans genome) and the dCas9 and other components of this plasmid. Results from our growth curve analysis indicated that strains harboring the dCas9 plasmid grew comparably to the wild-type strains and achieved the same maximum cell density, as monitored by optical density (OD_600_) (Fig. 1c). We subsequently used this dCas9 strain with nontargeting sgRNA as the wild-type control strain for future experiments. This further validates the utility of the dCas9-based CRISPRi system for genetic repression in C. albicans.

### Optimization of CRISPRi for genetic repression in C. albicans.

**FIG 2:**
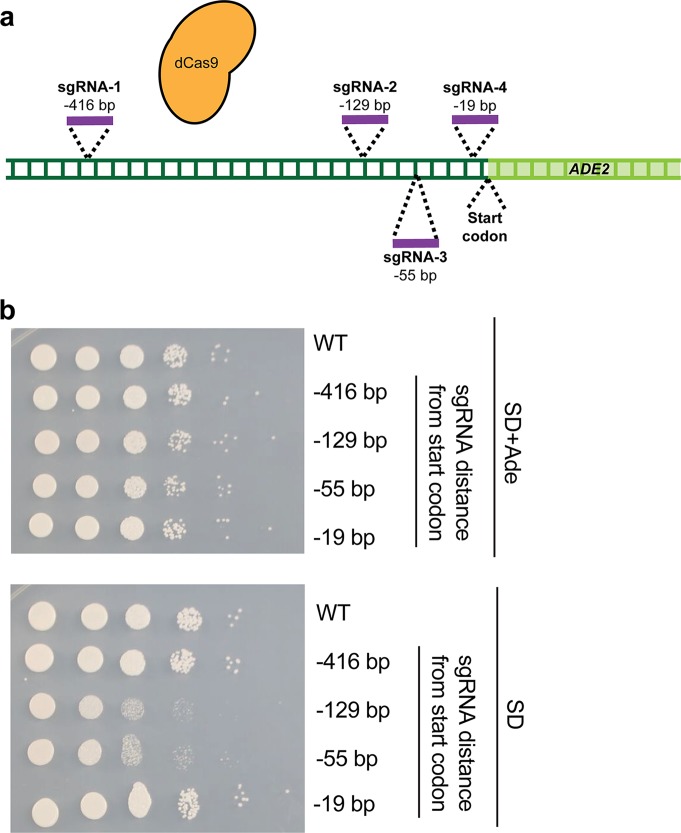
Optimization of CRISPRi for genetic repression in C. albicans. (a) Promoter region of the *ADE2* gene targeted with sgRNAs. Four sgRNAs were designed at four distinct loci upstream of the *ADE2* start codon (−416, −129, −55, and −19 bp upstream). (b) Identifying a promoter region for CRISPRi targeting. C. albicans strains were generated, each of which contained a dCas9 plasmid and one of the four sgRNAs described for panel a. To determine the extent of *ADE2* repression, growth was monitored by serial dilution spotting assays on SD media with or without supplemented adenine (Ade). Two strains (those with −129-bp and −55-bp sgRNAs) showed reduced growth on SD medium without adenine, suggesting that those strains successfully repressed *ADE2*.

Next, we aimed to assess whether our CRISPRi system could achieve transcriptional repression of the genes of C. albicans. We further aimed to assess which region of a promoter would be optimal for targeting the sgRNA-dCas9 complex in order to achieve maximal transcriptional repression. This was critical, as previous studies have found significant variability in CRISPRi-based repression levels, depending on the region targeted by dCas9 (47, 52). In order to determine if CRISPRi could repress transcription in C. albicans and the optimal targeting locus, we designed a CRISPRi system targeting the endogenous *ADE2* gene as a reporter. We designed four unique sgRNA N20s, targeting regions 416 bp (sgRNA-1), 129 bp (sgRNA-2), 55 bp (sgRNA-3), and 19 bp (sgRNA-4) upstream of the *ADE2* start codon, respectively (Fig. 2a). sgRNAs 1, 2, and 4 mapped to the sense DNA strand, while sgRNA 3 mapped to the antisense strand. Each of these four N20 sequences was cloned into the dCas9 backbone (Fig. 1a) to generate four unique plasmids targeting different regions upstream of *ADE2* for CRISPRi-based repression, and these constructs were used to generate four CRISPRi-*ADE2* C. albicans strains.

In order to monitor repression of *ADE2* transcription, we monitored growth of these four C. albicans strains, compared to growth of a wild-type control strain, on media containing or lacking adenine, as strains with depleted levels of *ADE2* should be impaired in their ability to grow in the absence of supplemented adenine. We performed this assay since we observed that, unlike genetic deletion or mutation of *ADE2*, transcriptional repression was not sufficient to render cells red. Results from serial dilution spotting assays on synthetic defined (SD) minimal media with or without supplemented adenine indicated that while all strains grew equally well on SD plus adenine, two of the CRISPRi *ADE2* depletion strains (those with sgRNAs targeting dCas9 129 bp and 55 bp upstream of the *ADE2* start codon) had impaired growth on medium lacking adenine (Fig. 2b). This suggests that our CRISPRi repression system is functional in C. albicans and is capable of repressing transcription from the endogenous *ADE2* locus, as indicated by the growth defect seen in the absence of supplemented adenine. It further indicates that, for *ADE2* repression, maximal transcriptional repression is achieved ∼55 to 129 bp upstream of the start codon and that CRISPRi is likely not strand specific in C. albicans, as both sense and antisense sgRNAs are capable of achieving repression (in agreement with what has been documented in S. cerevisiae [61]). This suggests important design principles for generation of additional CRISPRi constructs in C. albicans.

### Enhanced CRISPRi repression with dCas9-repressor fusion constructs.

**FIG 3:**
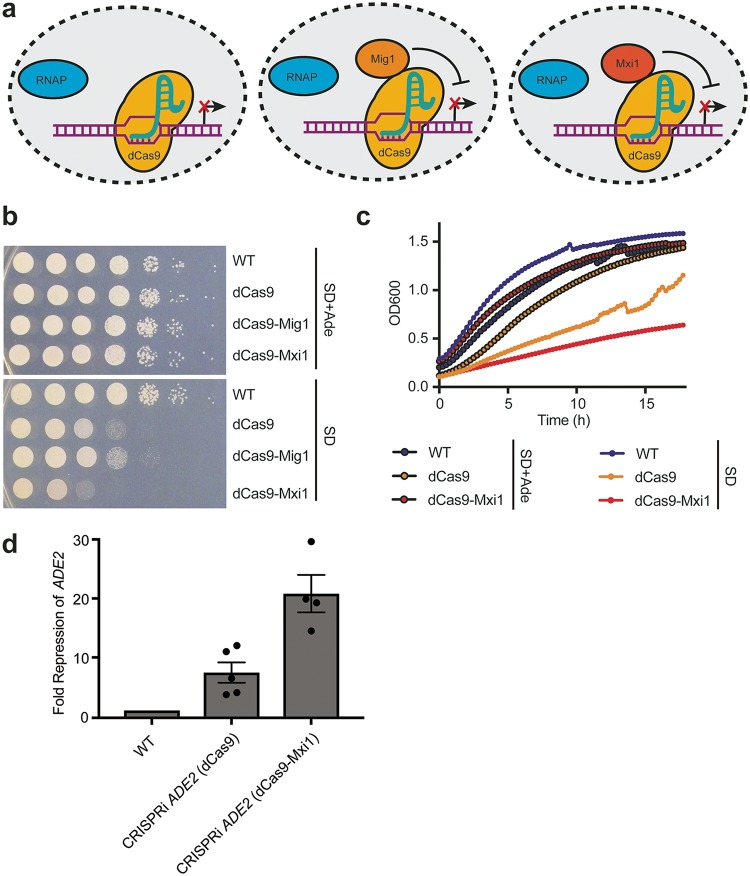
CRISPRi repression with dCas9-repressor fusion constructs. (a) dCas9 fusion constructs for CRISPRi repression. The diagram depicts the three dCas9 constructs (dCas9, dCas9-Mig1, and dCas9-Mxi1) engineered for CRISPRi repression in C. albicans. sgRNAs 1, 2, and 4 are on the sense strand; sgRNA 3 is antisense. RNAP, RNA polymerase. (b) dCas9-Mxi1 enhances repression from the *ADE2* locus. C. albicans strains were generated, with each containing a dCas9 plasmid (dCas9, dCas9-Mig1, or dCas9-Mxi1), each with the same sgRNA targeting *ADE2*. To determine the extent of *ADE2* repression, growth was monitored by serial dilution spotting assays on SD media with or without supplemented adenine. While dCas9 and dCas9-Mig1 showed reduced growth on SD without adenine medium, dCas9-Mxi1 showed further growth reduction, suggesting that this construct most effectively repressed *ADE2* expression. (c) Growth curves confirming dCas9-based repression from the *ADE2* locus. Wild-type, dCas9, and dCas9-Mxi1 C. albicans strains were grown in liquid SD media with or without supplemented adenine, and growth kinetics were monitored over ∼18 h. Both CRISPRi strains showed reduced growth in the absence of adenine, and the dCas9-Mxi1 strains showed further growth reduction. (d) qRT-PCR confirmed the reduced *ADE2* transcript levels. To validate the transcriptional repression via CRISPRi, qRT-PCR was performed on wild-type, dCas9, and dCas-Mxi1 strains. *ADE2* transcripts were monitored and normalized to an *ACT1* transcript as a housekeeping gene. Data were plotted as fold repression of *ADE2* relative to the wild-type control strain. Error bars depict standard errors of the means (SEM).

Since we were able to demonstrate transcriptional repression from the *ADE2* locus using a simple dCas9 CRISPRi construct in C. albicans, we next wanted to assess whether we could enhance transcriptional repression by fusing dCas9 to repressor domains. We chose two transcriptional repressors for dCas9 fusion: (1) Mxi1, a mammalian transcriptional repressor domain, previously reported to enhance CRISPRi-based repression in S. cerevisiae (52) and suggested to interact with the yeast histone deacetylase and transcriptional repressor Sin3 (52, 62); and (2) Mig1, a well-characterized S. cerevisiae transcriptional repressor protein (63) that has also been demonstrated to enhance CRISPRi repression in S. cerevisiae (64). Therefore, we designed C. albicans codon-optimized versions of Mxi1 and Mig1 and engineered two additional dCas9 CRISPRi plasmids with Mxi1 or Mig1 fused to C-terminal end of dCas9 (Fig. 3a; see also Fig. S1 in the supplemental material).

10.1128/mSphere.00002-19.1 FIG S1 Engineered dCas9 fusion plasmids for CRISPRi repression. These dCas9-based plasmids are designed for CRISPRi repression in C. albicans based on dCas9 fusion to repressor Mig1 or Mxi1. All components have been codon optimized for C. albicans. *NEUT5L* homology is present for integration into the C. albicans genome upon plasmid linearization with PacI. The two SapI cloning sites allow simple sgRNA N20 cloning for generation of unique sgRNAs. Download FIG S1, TIF file, 1.1 MB. Copyright © 2019 Wensing et al. 2019 Wensing et al. This content is distributed under the terms of the Creative Commons Attribution 4.0 International license.

In order to determine if the dCas9, dCas9-Mxi1, and dCas9-Mig1 constructs would be able to repress expression to various degrees, we cloned the sgRNA N20 targeting 129 bp upstream of the *ADE2* promoter into these plasmids. We then transformed these constructs into C. albicans to generate strains with three unique CRISPRi constructs, each targeting the same *ADE2* locus for repression (Fig. 3a; see also Fig. S1). We monitored repression of *ADE2* using serial dilution spotting assays on SD minimal media with or without adenine and confirmed that the dCas9 construct was able to repress *ADE2* expression, based on reduced growth in the absence of supplemented adenine (Fig. 3b). The dCas9-Mig1 strain demonstrated reduced growth in the absence of adenine to an extent similar to that seen with the dCas9 strain, while the dCas9-Mxi1 strain showed significantly reduced growth in the absence of adenine, suggesting that this strain was able to repress expression of *ADE2* most effectively (Fig. 3b). Two independently generated dCas9-Mxi1 strains were tested for *ADE2* repression via growth on medium lacking adenine, and the two demonstrated the same phenotype (data not shown).

To further confirm this finding, we monitored growth kinetics of wild-type, dCas9, and dCas9-Mxi1 C. albicans strains in liquid SD minimal media with or without adenine over 18 h and confirmed that both the dCas9 and dCas9-Mxi1 strains were impaired in growth in the absence of adenine, suggesting that *ADE2* was repressed in those CRISPRi strains (Fig. 3c). And, similarly to what we observed in serial dilution spotting assays, the dCas9-Mxi1 strain grew less well than the dCas9 strain, suggesting that this strain achieved higher levels of *ADE2* repression (Fig. 3c). Finally, to quantify the level of transcriptional repression achieved in the C. albicans dCas9 and dCas9-Mxi1 strains, we used quantitative reverse transcription-PCR (qRT-PCR) to monitor the relative expression levels of *ADE2* in these strains. We found that the dCas9 strain was able to achieve ∼7-fold repression of *ADE2*, while the dCas9-Mxi1 strain showed ∼20-fold repression of *ADE2* (Fig. 3d). Taking the data together, this suggests that the dCas9-Mxi1 fusion construct will be a valuable tool to efficiently repress transcription of genes in C. albicans.

### CRISPRi*-*mediated repression of an essential C. albicans gene.

Finally, we validated the use of this dCas9-Mxi1 CRISPRi platform for repression of an essential gene, since the ability to target essential genes is a significant advantage of using CRISPRi technology. For this analysis, we chose the essential molecular chaperone Hsp90, which has been well characterized in C. albicans and is known to be involved in both cellular morphogenesis and resistance to antifungal drugs (65–67). Therefore, we developed two CRISPRi constructs, each with a distinct sgRNA, targeting −141 bp (sense strand sgRNA) and −112 bp (antisense strand sgRNA) upstream of the *HSP90* start codon, respectively. These regions were chosen based on our optimization with *ADE2* and were cloned into the dCas9-Mxi1 plasmid backbone. We used these two CRISPRi constructs to generate two C. albicans mutant strains, each containing one of the *HSP90* CRISPRi repression constructs.

**FIG 4:**
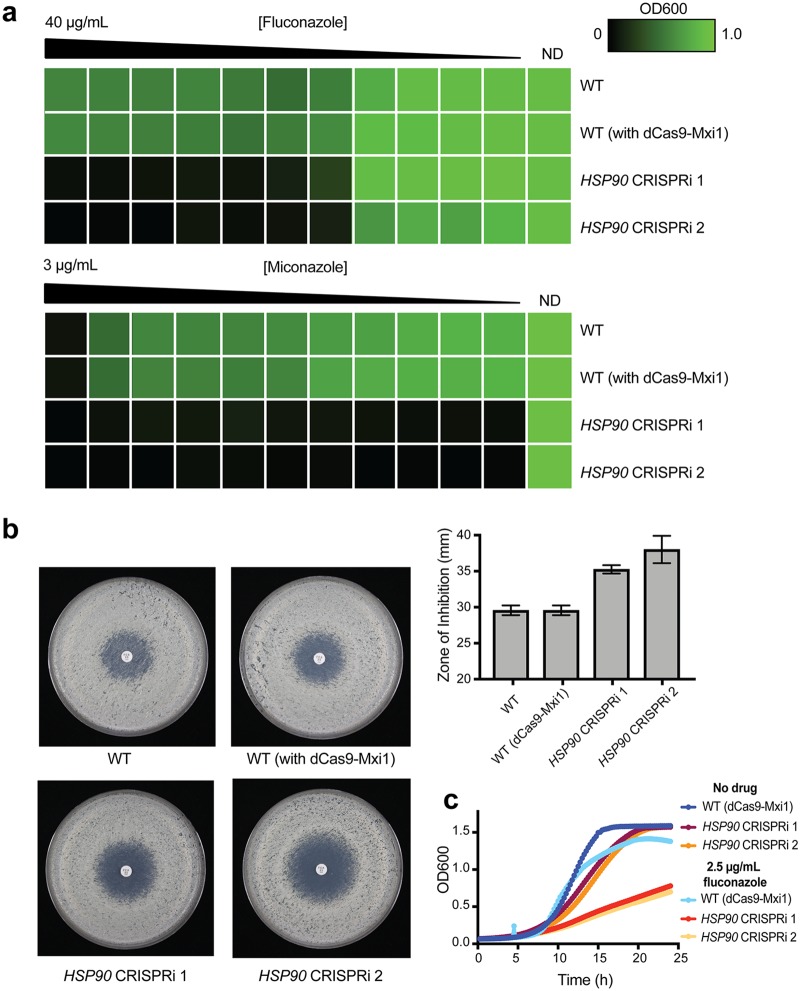
CRISPRi-based repression of the essential gene *HSP90* in C. albicans. (a) Reduced levels of *HSP90* rendered C. albicans more sensitive to azoles in MIC assays. MIC assays were performed with a gradient of an azole drug, namely, fluconazole (from 40 µg/ml to 0 µg/ml) or miconazole (from 3 µg/ml to 0 µg/ml), in 2-fold serial dilutions. Growth of all strains was monitored across the drug gradients and in a no-drug control (ND). The strains tested for antifungal susceptibility are indicated as follows: WT (*SC5314*), WT (with dCas9-Mxi1) (strain containing only dCas9-Mxi1 with nontargeting sgRNA integrated at the *NEUT5L* locus [also known as fRS187]), and *HSP90* CRISPRi 1 (fRS221) and *HSP90* CRISPRi 2 (fRS222) (two independently generated *HSP90* CRISPRi dCas9-Mxi1 strains, each with a unique sgRNA targeting *HSP90* for repression). Growth was normalized relative to the no-drug control, and data were quantitatively visualized using TreeView3. (b) Reduced levels of *HSP90* render C. albicans more sensitive to fluconazole in disk diffusion assays. Disk diffusion assays were performed using a 25-µg-fluconazole disk on Casitone agar plates. Growth of wild-type (WT) strains (including *SC5314* WT and a WT strain containing only dCas9-Mxi1 with nontargeting sgRNA at the *NEUT5L* locus) and of two independent *HSP90* CRISPRi dCas9-Mxi1 strains (each with a unique sgRNA targeting *HSP90* for repression) was observed on these plates after 24 h and 48 h (48-h results are depicted here). Quantification of the zone of inhibition (measured using the *diskImageR* program [87]) is depicted in the graph. (c) Growth curves confirming sensitivity of *HSP90* depletion strains. The dCas9-Mxi1 wild-type strain and both *HSP90* CRISPRi strains of C. albicans were grown in liquid YPD media with no drug or with 2.5 µg/ml fluconazole, and growth kinetics were monitored over ∼25 h. Both CRISPRi strains showed reduced growth in the presence of fluconazole.

To validate repression of *HSP90* in these mutant C. albicans strains, we assessed phenotypes known to be associated with repression of *HSP90* in CRISPRi mutant strains compared with wild-type control strains. We monitored resistance to the azole antifungals fluconazole and miconazole, as repression of *HSP90* has been shown to abrogate resistance to these drugs (66, 68). We performed MIC assays with fluconazole and miconazole and found that, using either of the two sgRNA constructs, CRISPRi-based repression of *HSP90* led to increased sensitivity to both azole drugs (Fig. 4a), as predicted based on previously observed phenotypes. We further used a fluconazole disk diffusion assay to confirm that repression of *HSP90* led to increased susceptibility to fluconazole, based on a larger zone of inhibition (Fig. 4b). Finally, we confirmed the enhanced fluconazole sensitivity of these *HSP90* CRISPRi strains by monitoring growth kinetics of these strains and of the corresponding wild-type strain, in the absence or presence of fluconazole (Fig. 4c). Taking the results together, this demonstrates that the *HSP90* CRISPRi strains show phenotypes that correspond with depletion of *HSP90*, and confirms the utility of this CRISPRi system for the effective genetic depletion of essential genes.

## DISCUSSION

Here, we demonstrated the first application of a CRISPRi-based genetic repression system in the human fungal pathogen C. albicans. We generated nuclease-dead Cas9 (dCas9) CRISPRi plasmids, optimized for use in C. albicans, with a simple sgRNA cloning site to enable efficient and rapid Golden Gate cloning of any sgRNA N20 at that locus, to target any gene of interest for repression. We further optimized a region for targeting sgRNA-dCas9, −55 to 129 bp upstream of the start codon, on the basis of an *ADE2* CRISPRi system. We note that while this promoter targeting region may vary for different C. albicans genes, it is similar to the optimal CRISPRi targeting region for S. cerevisiae, which is between ∼0 to 200 bp upstream of the transcription start site (54). We further engineered novel CRISPRi fusion constructs for use in C. albicans and demonstrated that dCas9, dCas9-Mig1, and dCas9-Mxi1 are each able to effectively repress gene expression in C. albicans, with the dCas9-Mxi1 fusion being the strongest repressor (∼20-fold gene repression). Finally, we demonstrated the capability of this system to target essential genes for genetic depletion, using *HSP90* as a candidate essential gene target. Taking the results together, we believe this CRISPRi system will be a powerful tool for efficient genetic repression in C. albicans, with many possible applications for functional genetic studies and for the analysis of essential genes.

This work represents the first CRISPRi system for use in C. albicans, and, to our knowledge, the first for use in any fungal pathogen. However, CRISPRi technologies have been developed as powerful tools to study many other organisms, including several important microbial species. Foundational CRISPRi studies demonstrated the application of this technology in E. coli (47, 69), and CRISPRi was subsequently applied to numerous other bacterial species. One important application of CRISPRi systems has been the study of essential genes, as CRISPRi enables partial loss of function through repression of these necessary genetic factors rather than a complete loss of function. In the Gram-positive model bacterium Bacillus subtilis, a CRISPRi screen was used to target all essential genes for systematic investigation of the phenotypes associated with these factors, revealing novel genetic networks and morphological phenotypes associated with essential genes (50). Similar CRISPRi-based gene knockdown screens were used to identify previously uncharacterized essential genes in Streptococcus pneumoniae (70). CRISPRi has also been applied in *Pseudomonas* species to study the function of essential genes involved in cell division (53) and to identify and study the roles of essential genes, and their interactions, in *Mycobacterium* species (51, 71). Similar CRISPRi studies in C. albicans or other fungal pathogens could help unravel the function of essential genes in these important microbial organisms.

Two valuable features of our C. albicans CRISPRi system are its efficiency and its potential for scalability. We have designed the dCas9 plasmid (and dCas9-fusion plasmids) to be a singular plasmid system, with a simple sgRNA cloning system, requiring only the synthesis of small, 20-bp N20 sequences. The cost-effectiveness of this system (requiring only two 23-bp oligonucleotides per gene being targeted for repression) and the proven efficiency of the Golden Gate cloning strategy suggest that this system could readily be scaled up to the genome level. Additionally, in C. albicans CRISPRi strains, the sgRNA can act as an inherent DNA barcode, containing both conserved regions (*SNR52* promoter, sgRNA tail) and strain-specific sequences (sgRNA N20). This could enable CRISPRi pooled screens of C. albicans strains similar to the CRISPRi pooled screens employed for genome-scale functional genomic analysis in E. coli (72). The use of such pooled competition assays among mutant microbial strains has been a powerful strategy for functional-genomic profiling and chemical-genomic analysis in S. cerevisiae (73–76) and C. albicans (12, 22), and CRISPRi could provide a complementary approach to further enable such studies.

Finally, the development of a functional CRISPRi system in C. albicans facilitates the application of other dCas9-based systems in this organism. Since we are able target dCas9 to targeted genetic loci through deliberate sgRNA design, we can further exploit this technology by fusing other effector domains to dCas9, as has been demonstrated in several other systems (46). As previously described, CRISPRa enables the activation of genes of interest (55–57, 77) and could be applied to our C. albicans system. Such overexpression systems could provide a platform for antifungal drug target identification (27), as demonstrated by the use of similar systems in S. cerevisiae (78). Further, CRISPR-based epigenetic modifications can be achieved through fusion of dCas9 to epigenetic regulators, such as histone demethylase or acetyltransferase enzymes (46). Such CRISPR-epigenetic systems have primarily been applied in mammalian systems (44, 45) but could similarly enable targeted epigenetic regulation in microbial organisms. Additionally, improvements to this C. albicans CRISPRi system could be made by fusing multiple repressor domains to dCas9—a strategy that has been used successfully to enhance repression in S. cerevisiae (64). Taking the results together, this work has generated a new tool to enable genetic repression in C. albicans with potential for adaptation for other CRISPR-based applications and for use in other related fungal pathogens.

## MATERIALS AND METHODS

### Strains and culture conditions.

Strains used in this study are listed in Table S1 in the supplemental material, and plasmids are listed in Table S2. C. albicans strains were cultured on YPD (2% Bacto peptone, 1% yeast extract, 2% glucose), and E. coli strains were cultured in LB media.

10.1128/mSphere.00002-19.2 TABLE S1 Strain table, representing a list of relevant strains used in this research. Download Table S1, XLSX file, 0.01 MB. Copyright © 2019 Wensing et al. 2019 Wensing et al. This content is distributed under the terms of the Creative Commons Attribution 4.0 International license.

10.1128/mSphere.00002-19.3 TABLE S2 Plasmid table, representing a list of relevant plasmids used in this research. Download Table S2, XLSX file, 0.01 MB. Copyright © 2019 Wensing et al. 2019 Wensing et al. This content is distributed under the terms of the Creative Commons Attribution 4.0 International license.

### Plasmid generation.

The plasmid backbone used in this study was adapted from the C. albicans-optimized CRISPR-Cas9 plasmid (also known as pRS252) used in our previous study (33), containing the *NEUT5L* homology site and *CAS9* (79). To create a sgRNA cloning locus in this plasmid, the *SNR52* promoter, SapI cloning locus, and sgRNA tail were synthesized *in vitro* as gBlocks gene fragments from Integrated DNA Technologies (IDT) and were cloned into the CRISPR-Cas9 plasmid (pRS252) at the NgoMIV restriction enzyme site, using Gibson assembly, as previously described (33, 79). We have made the relevant CRISPRi (dCas9 and dCas9-Mxi1) plasmids available via Addgene (reference numbers 122377, 122378, 122379, and 122380).

### Site-directed mutagenesis.

Two nuclease mutations were introduced into Cas9 (D10A and N863A) to render it nuclease-dead (dCas9). These targeted mutations designed to disrupt Cas9 catalytic activity were introduced using site-directed mutagenesis as previously described (80).

### dCas9 fusion construction.

dCas9 fusion proteins were generated through the use of Gibson assembly. The Mxi1 effector domain was codon optimized for C. albicans expression and synthesized *in vitro* as gBlocks gene fragments from IDT, while the Mig1 coding sequence was directly amplified from C. albicans genomic DNA. These fragments were then cloned with Gibson assembly into the dCas9 plasmid backbone. The Mig1 gene and the Mxi1 domains were selected based on previous publications (52, 81).

### sgRNA design.

sgRNA N20 sequences were designed based on an efficiency score and predicted specificity using the C. albicans genetic sequences from the *Candida* Genome Database (CGD; http://www.candidagenome.org) (82) and the sgRNA design tool Eukaryotic Pathogen CRISPR gRNA Design Tool (EuPaGDT) (83) available at http://grna.ctegd.uga.edu.

### sgRNA Golden Gate cloning.

sgRNA N20 sequences were cloned into the dCas9 plasmid at the sgRNA cloning locus (containing the *SNR52* promoter, SapI cloning locus, and sgRNA tail) using Golden Gate cloning (60), as previously described (79). Each sgRNA N20 sequence was obtained as two oligonucleotides from IDT in forward and reverse complement orientation. Each of the two complementary oligonucleotides contained a SapI cloning site, and each was reconstituted to 100 µM using nuclease-free duplex buffer from IDT. Equal volumes of the two complementary oligonucleotides were then combined and duplexed together by heating to 94°C for 2 min and allowed to cool to room temperature. To clone the duplexed fragment into the dCas9 plasmid, the following were combined: 10 µl miniprepped dCas9 plasmid, 1 µl duplexed oligonucleotide, 2 µl 10× CutSmart buffer, 2 µl ATP, 1 µl SapI, 1 µl T4 DNA ligase, and 3 µl nuclease-free water. This mixture was incubated in a thermocycler under the following cycling conditions: (37°C, 2 min; 16°C, 5 min) for 99 cycles; 65°C, 15 min; 80°C, 15 min. After cycling was complete, 1 µl of additional SapI enzyme was added to each reaction mixture, and the mixture was incubated at 37°C for 1 h.

### Bacterial transformation.

Golden Gate-ligated plasmids were transformed into chemically competent DH5α Escherichia coli cells. Competent cells (50 µl) were combined with 5 µl plasmid and incubated on ice for 30 min, heat shocked at 42°C for 30 s, and then incubated on ice for 5 min. This mixture was then added to 950 µl of Super Optimal Broth with added glucose (SOC media) and incubated at 37°C for 1 h with shaking. Transformed cells were selected on LB plates containing 100 µg/ml ampicillin.

### Plasmid PCR validation.

Ampicillin-resistant bacterial colonies were genotyped by colony PCR to confirm proper integration of the sgRNA N20 in the dCas9 plasmid. Briefly, bacterial colonies were diluted in 100 µl of nuclease-free water, and 5 µl of the mixture was added to a PCR with 2× *Taq* polymerase mix and oligonucleotide primers. For each PCR, the primer pair was in the forward orientation N20 oligonucleotide plus TATACCATCCAAATCAATTCC and in the reverse complement orientation N20 oligonucleotide plus ACCCACTGAATTCTACATCGAAC. PCRs were run on 1% agarose gels.

### C. albicans transformation.

All C. albicans strains were generated using a lithium acetate transformation protocol, as previously described (79). Briefly, dCas9 plasmids were linearized with PacI restriction enzyme. Linearized plasmid and C. albicans cells were incubated with 800 µl 50% polyethylene glycol (PEG), 100 µl 10× Tris-EDTA (TE) buffer, 100 µl 1 M lithium acetate (pH 7.4), 40 µl of salmon sperm DNA, and 20 µl 2 M dithiothreitol (DTT). This mixture was then incubated at 30°C for 1 h and at 42°C for 45 min. Cells were grown in YPD media for 4 h at 30°C with shaking and were then selected for on YPD plates containing 200 µg/ml nourseothricin (NAT).

### C. albicans PCR validation.

NAT-resistant bacterial colonies were genotyped by colony PCR to confirm proper integration of the dCas9 plasmids at the *NEUT5L* locus. Briefly, C. albicans colonies were diluted in 100 µl of nuclease-free water, and 5 µl of this was added to a PCR with 2× *Taq* polymerase mix and oligonucleotide primers. For each PCR, primers ACTATTAAAGAACGTGGACTCCAACGTCA (in the dCas9 plasmid) and CAAGTTTGCACTCTTTTGTCTA (in the genomic *NEUT5L* locus) were used to validate integration. PCRs were run on 1% agarose gels.

### Serial dilution spotting assays.

C. albicans overnight cultures were diluted in 10-fold serial dilutions in sterile phosphate-buffered saline (PBS) media, and 5 µl of each diluted culture was spotted onto synthetic defined (SD; 0.67% yeast nitrogen base without amino acids, 2% glucose) agar plates with or without supplemented adenine.

### Growth curve assays.

C. albicans cultures were grown overnight in YPD media. Cells were diluted to an OD_600_ of 0.05 in 96-well microtiter plates and grown at 37°C with continuous shaking, using a PerkinElmer Victor microplate reader or Bio-Rad xMark plate reader. For adenine growth curve assays, strains were grown in SD medium with or without supplemented adenine. For fluconazole growth curve assays, strains were grown in the absence of drug or with 2.5 µg/ml fluconazole–YPD. Each strain was grown in 3 or 4 independent wells. Optical density was measured at 600 nm every 15 min over 18 to 25 h.

### Quantitative reverse transcription-PCR (qRT-PCR).

To monitor *ADE2* transcript levels, qRT-PCR was performed as previously described (84). Briefly, C. albicans cells were grown overnight in YPD at 37°C, diluted to an OD_600_ of 0.2, and grown to an OD_600_ of ∼1.0 at 37°C. Cultures were then pelleted and frozen overnight at –80°C. RNA was isolated using a Geneaid yeast total RNA minikit supplemented with zymolyase. cDNA synthesis was performed using 800 ng RNA and a High Capacity cDNA reverse transcription kit (Applied Biosystems). PCR was performed using 2× PerfeCta SYBR green FastMix from Quanta BioScience under the following cycling conditions: 30 s at 95°C for the polymerase activation step, followed by 40 cycles of a two-step quantitative PCR (qPCR) procedure (3 s of 95°C denaturation, 30 s of 60°C combined annealing/extension). The primers used were as follows: for *ADE2*, TTAGTGTATGCTCCTGCCAGG and GAGTTGTGAGGTCTTGGTGC; for *ACT1* (control), GTTGGTGATGAAGCCCAATCC and CTGGATGTTCTTCTGGAGCAAC.

### MIC assays.

MIC assays were performed in 96-well microtiter plates, according to a standard broth microdilution protocol (85), with some modifications. Briefly, MIC tests were set up in a total volume of 200 µl per well with 2-fold serial dilutions of fluconazole or miconazole in YPD media. The gradients of fluconazole were 40 to 0 µg/ml, and the gradients of miconazole were 3 to 0 µg/ml, in 2-fold dilutions. The strains used for MIC analysis were grown overnight in YPD at 30°C. The cell densities of overnight cultures were determined by optical density (OD_600_), and dilutions were prepared such that equal numbers of cells were inoculated into all wells. MIC plates were incubated at 37°C for 24 to 48 h. After incubation, the optical density of cells in each well was determined at 600 nm using a microplate reader (PerkinElmer Victor) and growth of each strain was normalized to growth in the absence of drug. Each strain was tested in duplicate on at least three independent occasions. MIC data were quantitatively displayed with color using the program TreeView3.

### Disk diffusion assays.

Antifungal disk diffusion analysis was assessed using modified CLSI-M44-A2 guidelines on disk diffusion susceptibility adapted from a previous study (86). Strains were grown overnight on YPD agar at 30°C and were then resuspended in 1.5 ml of filter-sterile PBS and diluted to an OD_600_ of 0.1. A 200-µl volume of each resuspended strain was spread onto 15-ml Casitone agar plates (9 g/liter Bacto Casitone, 5 g/liter yeast extract, 11.5 g sodium citrate dehydrate, 20 g/liter glucose, 15 g/liter Bacto agar) via glass bead spreading. One 25-μg fluconazole disk (Oxoid, United Kingdom) (6 mm in diameter) was placed at the center of each plate. The plates were then incubated at 30°C, and photographs were taken after 24 and 48 h. Each strain was tested on duplicate plates, on at least three independent occasions. The computational pipeline *diskimageR* was used to assess the results of the antifungal diffusion assay (87).

### Data availability.

The relevant CRISPRi (dCas9 and dCas9-Mxi1) plasmids are available via Addgene under reference numbers 122377, 122378, 122379, and 122380.
