# Supplementary figures and images for: A CRISPR Interference Platform for Efficient Genetic Repression in Candida albicans
Source: mSphere. 2019 Feb 13;4(1):e00002-19. doi: 10.1128/mSphere.00002-19 (PMC6374589; doi:10.1128/mSphere.00002-19)

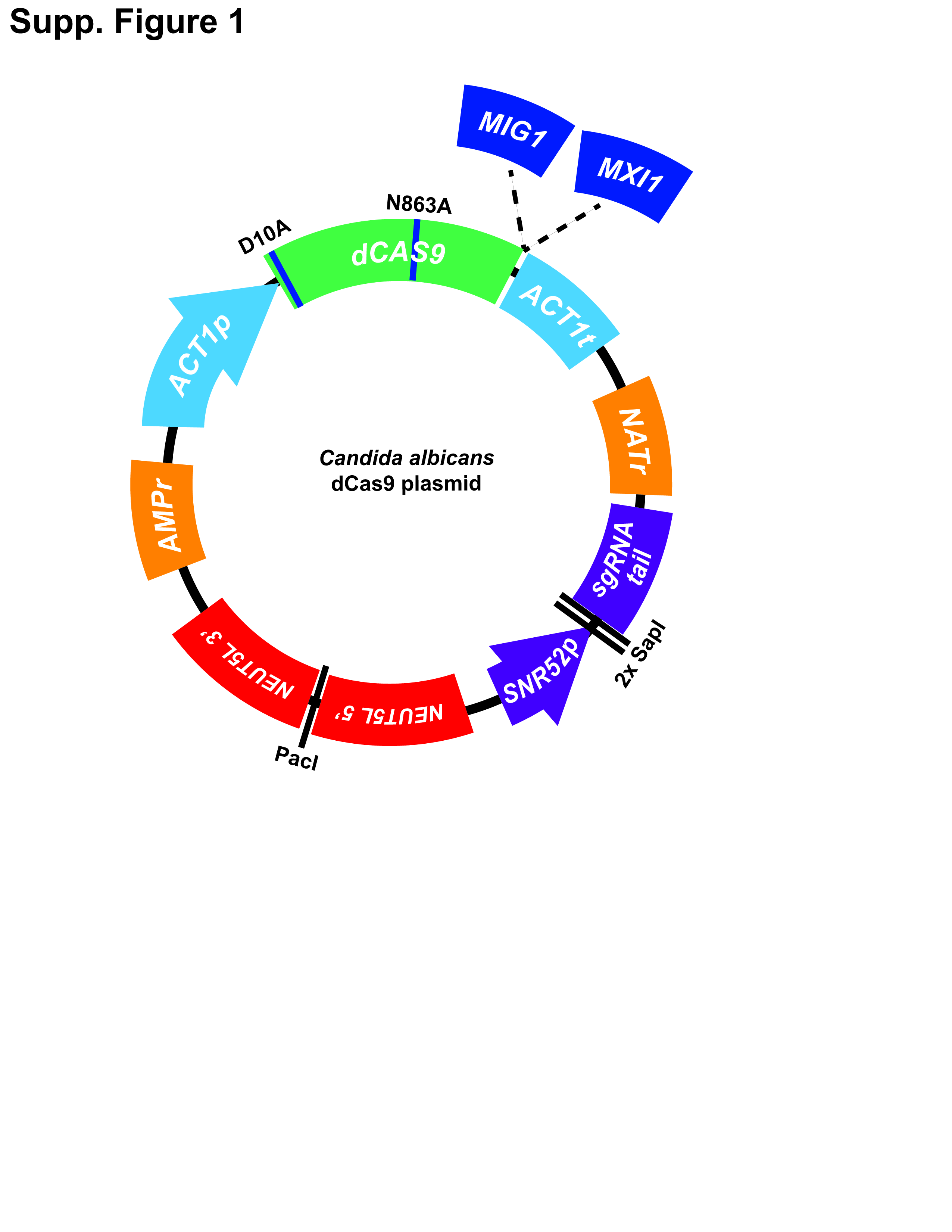

Supplement: FIG S1 [file mSphere.00002-19-sf001.tif]
